# Supplementary material for: Development of an interactive, agent-based local stochastic model of COVID-19 transmission and evaluation of mitigation strategies illustrated for the state of Massachusetts, USA
Source: PLoS One. 2021 Feb 17;16(2):e0247182. doi: 10.1371/journal.pone.0247182 (PMC7888623; doi:10.1371/journal.pone.0247182)
Supplement: S1 Appendix — The details about the model formulation, parameterization, and calibration. (PDF) [file pone.0247182.s001.pdf]

Development of an interactive, agent-based local stochastic model of COVID-19 transmission and evaluation of mitigation strategies illustrated for the state of Massachusetts, USA

Alexander Kirpich[1,2◑], Vladimir Koniukhovskii[3◑], Vladimir Shvartc[3], Pavel Skums[4], Thomas A. Weppelmann[5], Evgeny Imyanitov[6], Semyon Semyonov[7], Konstantin Barsukov[3], Yuriy Gankin[7*]

1 Department of Population Health Sciences, Georgia State University, Atlanta, Georgia, United States
2 School of Public Health, Georgia State University, Atlanta, Georgia, United States
3 EPAM Systems, Saint Petersburg, Russia
4 Department of Computer Science, Georgia State University, Atlanta, Georgia, United States
5 Department of Internal Medicine, University of South Florida, Tampa, Florida, United States
6 N.N. Petrov Research Institute of Oncology, Saint Petersburg, Russia
7 Quantori, Cambridge, Massachusetts, United States

◑These authors contributed equally to this work.
* yuriy.gankin@quantori.com

S1 Appendix

The Model Structure

The model stochastic simulations are generating the infected individuals at different times and stages. Those individuals are indexed by k and have individual characteristics presented below:

$$Q_k = \Big((x_k, y_k), tinf_k, det_k, stg_k, age_k, rad_k, p_{cont(k)}, cont_k, R_{0(k)}, sever_k, dur_k, st_k(t)\Big). \quad (1)$$

The characteristics of each individual Q_k are determined and updated within the simulation process and have the following details:

- (x_k, y_k) - the Cartesian coordinates (in pixels) of the individual Q_k that do not change within the simulation process after they are introduced;
- $tinf_k$ – the time of infection onset for the individual Q_k which is measured in relation to the simulation baseline time denoted as 0;
- det_k – the detection time variable measured in days that corresponds to a period from an infection acquisition until the proper diagnosis and reporting of individual Q_k;
- $stg_k = \big(stg_{1(k)},\ stg_{2(k)},\ stg_{3(k)}\big)$ – the vector of durations of three disease infection stages measured in days that characterize the infectivity of the given individual during those stages. It is assumed that $stg_{1(k)} + stg_{2(k)} + stg_{3(k)} = det_k$;

- age_k – the age of the individual Q_k at the time of the infection onset;
- rad_k – the distance (in meters) up to which the individual Q_k is able to infect the nearby individuals;
- $p_{cont(k)}$ – the probability that during each day the individual Q_k has any contacts which lead to new infections;
- $cont_k = \left(\mu_{cont(k)}, \sigma^2_{cont(k)}\right)$ – the individual-specific parameters that define the distribution of the number of successful infection transmissions to other individuals within a given day. This number is generated randomly for each day t, provided that the individual has any transmissions on the given day (according to the contact probability $p_{cont(k)}$).
- $R_{0(k)}$ – the individual's reproduction number. This variable stores the number of individuals that are infected by Q_k during the infection period det_k. The average of those reproduction numbers across individuals and simulations are used to estimate the basic reproduction number $\mathcal{R}_0$ [1] which is a characteristic of the entire epidemic;
- $sever_k$ – the disease severity variable for the individual Q_k that takes three values, where 1 corresponds to lethal, 2 corresponds to severe, and 3 corresponds to mild; the disease severity does not change for a given individual after it is determined randomly from a trinomial distribution;
- dur_k – the disease duration from the infection onset to cure (or death) in days, which is generated randomly based on the $sever_k$ parameter;
- $st_k(t)$ - the status of the individual Q_k at a given day t. The status of the individuals within the simulation is expected to change over time and is expected to take the following values:
  - $st_k(t) = 0$ – the individual is detected based on the external information i.e. from the reported data that are used as the model input;
  - $st_k(t) = 1$ - the individual is infected but has not been identified as such yet;
  - $st_k(t) = 2$ – the individual has been infected and detected as such, which has also implied the individual's isolation (quarantine).
  - $st_k(t) = 3$ – the individual has recovered and is immune;
  - $st_k(t) = 4$ – the individual has deceased.

The asymptomatic infections are incorporated inside the model via three severities that are generated for each infected individual. This incorporation of asymptomatic infections into those with a "mild" severity status is made to improve the model computational tractability. The first severity status is "mild" which incorporates asymptomatic individuals as well. Then after an individual within this undetected class with any severity status is getting detected (i.e. tested and confirmed to have an infection) this individual is assigned the "detected" status ($st_k(t) = 1$) and is immediately quarantined. This approach allows to incorporate all infected individuals with different disease severities into the infected status and to isolate the confirmed cases. Since the model parameters are calibrated based on the detected cases and the output of the model presents the detected cases as well the prediction abilities for detected and reported cases within the model are preserved.

In the beginning of the simulations the model utilizes multiple local epidemic epicenters $\boldsymbol{E} = \{E_1, E_2, \ldots, E_{\mathcal{I}}\}$. Those epicenters serve as the model initial conditions

and represent the geographic points for the index cases that are introduced into the susceptible population. The epicenters can either correspond to the actual address coordinates for those places where the initial outbreaks were detected or to the centers of the corresponding aggregated geographic units. The latter may be the case, if either the exact infection acquisition locations are not known, or the privacy concerns prevent the inclusion of such data into the model. In the latter case the centers of the aggregated geographic units are taken as epicenters E_i for each $i = 1, 2, \ldots, \mathcal{I}$.

The local epicenters in the model are defined by a pair of geographic coordinates $(Lat, Long)$ and by an epicenter-specific region radius R_i which is defined in meters. Therefore, for $i = 1, 2, \ldots, \mathcal{I}$ the epicenter region is defined by a triplet:

$$E_i = ((Lat_i, Long_i), R_i)\,. \tag{2}$$

The epicenter regions are defined from the surveillance epidemiological data. As the initial conditions in addition to the local epicenters the model incorporates the areas of high density $\boldsymbol{P} = \{P_1, P_2, \ldots, P_{\mathcal{J}}\}$ for $j = 1, 2, \ldots, \mathcal{J}$, where each P_j represents a large city or a densely populated area and which is also defined by a triplet:

$$P_j = ((Lat_j, Long_j), R_j)\,. \tag{3}$$

In the model the reporting times (days) for the initial index cases for each epicenter i precede the modeled epidemic starting time which corresponds to the baseline time slot $t = 0$. Therefore, the reported time slot indexes across the epicenters E_i are denoted as $s = 1, 2, \ldots, \mathcal{S}$ with the corresponding times $\tilde{t}_1, \tilde{t}_2, \ldots, \tilde{t}_{\mathcal{S}}$. The earliest reported cases and their dates are used for the model input with indexes $s = 1, 2, \ldots, \tilde{\mathcal{S}}$ such that $\tilde{\mathcal{S}} < \mathcal{S}$ and the corresponding times $\tilde{t}_1, \tilde{t}_2, \ldots, \tilde{t}_{\tilde{\mathcal{S}}}$. The corresponding number of confirmed and reported infections for each local epicenter E_i up to and including the time $\tilde{t}_{\tilde{\mathcal{S}}}$ for $s = 1, 2, \ldots, \tilde{\mathcal{S}}$ is denoted as $n_i(\tilde{t}_{\tilde{\mathcal{S}}})$. The corresponding set of infected and reported (i.e. with the status $st_k(t) = 1$) individuals across all epicenter is denoted as:

$$\boldsymbol{D} = \left\{\tilde{Q}_1, \tilde{Q}_2, \ldots, \tilde{Q}_{\mathcal{K}_{\tilde{\mathcal{S}}}}\right\}, \tag{4}$$

where $k = 1, 2, \ldots, \mathcal{K}_{\tilde{\mathcal{S}}}$ is the global index for initial cases across all times $\tilde{t}_1, \tilde{t}_2, \ldots, \tilde{t}_{\mathcal{S}}$ and $\mathcal{K}_{\tilde{\mathcal{S}}}$ is the total number of the initial *index cases* simulated within the model based on the input data. The tilde notation for $\tilde{Q}_k$-s in $\boldsymbol{D}$ emphasizes the link to the model input data.

The time index that corresponds to individual day within the model is denoted as t and is equal to 0 at the model baseline. The simulation baseline time $t = 0$ corresponds to the latest reporting time $\tilde{t}_{\tilde{\mathcal{S}}}$ of the earliest reported cases that are used for the model input. The actual infection times for those index cases precede the selected baseline simulation time $t = 0$ due to the infectivity periods generated for those index cases prior to their reporting. The actual simulation starting time that accounts for the infectivity periods is denoted as $t = T_{min}$ and is smaller than the baseline time $t = 0$. This simulation starting time $t = T_{min}$ is generated within the model, while the baseline time $t = 0$ is defined by the data and is defined by the largest index within the set of calibration indexes $s = 1, 2, \ldots, \tilde{\mathcal{S}}$. The largest simulation time $t = T_{max}$ is determined by the model user based on the desired length of prediction. The initial set of index cases $\boldsymbol{D}$ from (4) defines the model initial cases that are allocated across the local epicenters (2) at times up to the baseline time $t = 0$.

Based on the model geographic characteristics (2) and (3) and the initial set of reported individuals $\boldsymbol{D}$ from (4) the new lists $\boldsymbol{\mathcal{L}}(t)$ of modeled individuals are simulated for time slots $t \in [T_{min}; T_{max}]$ where $T_{max} - T_{min} + 1$ is the total number of the simulated time slots. The simulated lists $\boldsymbol{\mathcal{L}}(t)$ have the following format:

$$\boldsymbol{\mathcal{L}}(t) = \left\{Q_1, Q_2, \ldots, Q_{\mathcal{K}(t)}\right\} \tag{5}$$

where the value of $\mathcal{K}(t)$ is defined by the simulation at every simulation time step $t \in [T_{min}; T_{max}]$. During this procedure the input set individuals $\boldsymbol{D}$ defined in (4) is allocated between the different epicenters and time slots within the lists $\boldsymbol{\mathcal{L}}(t)$ defined by (5). The allocations of the set $\boldsymbol{D}$ is performed during the time slots $t \in \left[T_{min}; \tilde{t}_{\tilde{\mathcal{S}}}\right]$ where $\tilde{t}_{\tilde{\mathcal{S}}} < T_{max}$.

Simulation of the Model Inputs Based on the Reported Data

The initial reported time point $\tilde{t}_1$ corresponds to the indexing time $t = -\tilde{\mathcal{S}}$ in relation to the selected baseline simulation time $t = 0$. For that time $\tilde{t}_1$ the cumulative number of infected and reported individuals at each epicenter E_i for $i = 1, 2, \ldots, \mathcal{I}$ is known from the model input and is denoted as $n_i(\tilde{t}_1) = n_i(-\tilde{\mathcal{S}})$. Those numbers are also assumed to correspond to the initial increase in cases during that first reported time $\tilde{t}_1$ i.e. it is assumed $\Delta n_i(\tilde{t}_1) = n_i(\tilde{t}_1)$ for each epicenter i. The subsequent time steps are indexed by $t = \tilde{t}_1 + \tau = -\tilde{\mathcal{S}} + \tau$ for the positive integer values of $\tau > 0$. Then the increase in the number of new cases that corresponds to index $t = -\tilde{\mathcal{S}} + \tau$ is defined as the difference in cumulative counts:

$$\Delta n_i(t) = \Delta n_i(\tilde{t}_1 + \tau) = \Delta n_i(-\tilde{\mathcal{S}} + \tau) = n_i(-\tilde{\mathcal{S}} + \tau) - n_i(-\tilde{\mathcal{S}} + \tau - 1).$$

In those notations for each epidemic epicenter $E_i \in \boldsymbol{E}$ at time index $t \in \left[-\tilde{\mathcal{S}}, \ldots, 0\right]$ the amount of the reported cases for the epicenter E_i is denoted as $\Delta n_i(t)$ and corresponds to the list:

$$\boldsymbol{D}_i(t) = \left\{\tilde{Q}_1, \tilde{Q}_2, \ldots, \tilde{Q}_{\Delta n_i(t)}\right\}. \quad (6)$$

In the end, the initially reported individuals from (6) for each epicenter E_i and timing index t are simulated as the model input in the following way:

- The geographic coordinates $(Lat_i, Long_i)$ of the epicenter E_i in the model are re-coded as Cartesian coordinates $C_i = (x_{i(c)}, y_{i(c)})$ which are defined in pixels. The character c in the subscript emphasizes that they belong to the epicenter. Since the modeled geographic region is relatively small in comparison to the entire planet surface, the discrepancies between the geographic and the Cartesian coordinate systems such as the differences in latitude scales within the region are negligible. The radius R_i (in peters) of each modeled circular epicenter in geographic coordinates is recalculated for the Cartesian coordinate system (in pixels) and is denoted as r_i.

  The initial numbers of infected individuals within each epicenter E_i is determined by the model input and those numbers are fixed. If the exact addresses and the corresponding geographic coordinates for individuals $\tilde{Q}_k$ are know, they can be imputed directly as points $M_k(x_k, y_k)$ in the Cartesian coordinate system within clusters E_i that they belong to. More often, however, due to privacy concerns only the geographic unit level data are known, and the individuals are reported for the geographic center of the administrative unit which is treated in the model as the center of E_i for some $i = 1, 2, \ldots, \mathcal{I}$. In this case the geographic locations of the reported individuals $\tilde{Q}_k$ within the given cluster E_i are randomly generated. The individual's coordinates $M_k = M_k(x_k, y_k)$ in the Cartesian coordinate system are generated in the following way:

  - The distance ρ_k from the center C_i to the generated point M_k is generated randomly from the exponential distribution with the scale parameter r_i i.e. $X \sim \mathcal{EXP}(r_i)$ with the density function:

    $$f(x) = \frac{1}{r_i} \exp\left[-\frac{x}{r_i}\right] \mathbb{I}\left[x \geq 0\right]$$

where r_i is the radius of the epicenter i.

– The polar angle φ_k of the point M_k in relation to the epicenter C_i is generated randomly from the uniform distribution defined on the closed interval that represents the unit radius circle i.e. from the $\mathcal{U}[0; 2\pi]$.

– The coordinates of the point M_k take the form:

$$x_k = x_{i(c)} + \rho_k \cos(\varphi_k) \text{ and } y_k = y_{i(c)} + \rho_k \sin(\varphi_k),$$

where the angle φ_k is counted counterclockwise in relation to the reference direction which corresponds to the x-axis direction in the Cartesian coordinate system.

- The values of the detection time det_k are generated from a log-normal distribution ($\mathcal{LN}$) with parameters $\left(\mu_{det}, \sigma^2_{det}\right)$ and the generated outcomes are rounded to integer values. The log-normal random variable X with parameters $\left(\mu_{det}, \sigma^2_{det}\right)$ is assumed to be generated by taking the value $X = \exp\left(\sigma_{det}\, Z + \mu_{det}\right)$ where Z is generated from the standard normal distribution (i.e. $Z \sim \mathcal{N}\left(0, 1\right)$). In this parameterization:

$$E[X] = \exp\left[\mu_{det} + \frac{\sigma^2_{det}}{2}\right] \text{ and } Var[X] = \left[\exp\left[\sigma^2_{det}\right] - 1\right] \cdot \exp\left[2\mu_{det} + \sigma^2_{det}\right].$$

This implies the formulas for μ_{det} and σ^2_{det} in terms of $Var[X]$ and $E[X]$:

$$\sigma^2_{det} = \ln\left[1 + \frac{Var[X]}{[E[X]]^2}\right] \text{ and } \mu_{det} = \ln\left[E[X]\right] - \frac{1}{2}\ln\left[1 + \frac{Var[X]}{[E[X]]^2}\right], \tag{7}$$

where ln stands for a natural logarithm function.

- For each index case $\tilde{Q}_k$ the infection time $tinf_k$ is defined as $tinf_k = \tilde{t}_s - det_k$ for some index $s \in \left[1, 2, \ldots \tilde{\mathcal{S}}\right]$ where $\tilde{t}_s$ corresponds to the time index when the individual $\tilde{Q}_k$ has been reported. The infection time $tinf_k$ for some individuals may be earlier than the time $\tilde{t}_1$ of the first reported case and the smallest of those times are taken as the actual beginning of the simulation T_{min} which is described in the simulation details.

- The lengths of the disease stages $stg_{1(k)}$ and $stg_{2(k)}$ for the individual $\tilde{Q}_k$ are generated from normal distributions with empirically calibrated parameters via the following sequence of steps. The random variable $S_{1(k)}$ is generated from the normal distribution $\mathcal{N}\left(0, (0.08 \cdot det_k)^2\right)$ and the length of first stage $stg_{1(k)}$ is defined as

$$stg_{1(k)} = \max\left\{\ \left[0.4 \cdot det_k + S_{1(k)}\right], 0\right\}, \tag{8}$$

which is subsequently rounded to an integer value. Then the random variable $S_{2(k)}$ is generated from the normal distribution $\mathcal{N}\left(0, \left(0.08 \cdot (det_k - stg_{1(k)})\right)^2\right)$ and the length of the second stage $stg_{2(k)}$ is defined as

$$stg_{2(k)} = \max\left\{\ \left[0.4 \cdot \left(det_k - stg_{1(k)}\right) + S_{2(k)}\right], 0\right\}, \tag{9}$$

which is subsequently rounded to an integer value. Finally, the duration of the last stage $stg_{3(k)}$ is defined as

$$stg_{3(k)} = det_k - stg_{1(k)} - stg_{2(k)}. \tag{10}$$

- The age age_k of the individual $\tilde{Q}_k$ is generated based on the empirical cumulative distribution function for the Massachusetts population is constructed from the 2020 state of Massachusetts Population Pyramid [2]. More precisely, the empirical cumulative distribution function is constructed from the pyramid as $F_{age}(a) := P([pyramid\ age] < a)$ for a given age variable a. The age of the individual $\tilde{Q}_k$ is generated as $age_k := F_{age}^{-1}(U)$ where U is a random variable generated from the standard uniform distribution (i.e. $U \sim \mathcal{U}[0;1]$).

- The value of the radius rad_k up to which the individual $\tilde{Q}_k$ is able to infect the nearby individuals is generated from a normal distribution with parameters $\mathcal{N}\left(\mu_{dist}, \sigma_{dist}^2\right)$. The value of the mean parameter μ_{dist} for the simulations of rad_k is multiplied by 0.7 and 0.6 for those individuals who are younger than 10 and older than 60 years since they are assumed to have more limited social mobility.

- The amount of contacts for every individual $\tilde{Q}_k$ for each day t is generated from a normal distribution with parameters from $cont_k$ i.e. from $\mathcal{N}\left(\mu_{cont(k)}, \sigma_{cont(k)}^2\right)$ with parameters that are specific to the individual $\tilde{Q}_k$. In particular, the mean distribution parameter $\mu_{cont(k)}$ is itself randomly generated once for each individual $\tilde{Q}_k$ and is adjusted in time based on the individual's disease state. The parameter $\mu_{cont(k)}$ is changed to $0.2 \cdot \mu_{cont(k)}$ during the latent period $stg_{1(k)}$, is equal to $\mu_{cont(k)}$ during the early symptomatic period $stg_{2(k)}$, and is changed to $1.5 \cdot \mu_{cont(k)}$ during the severe infection period $stg_{3(k)}$. The value of $\mu_{cont(k)}$ is generated from a normal distribution with parameters $\mathcal{N}\left(\mu_{dense}, \sigma_{dense}^2\right)$ if the individual's coordinates M_k belong to any area of high density P_j for some $j = 1, 2, \ldots \mathcal{J}$, and from a normal distribution with parameters $\mathcal{N}\left(\mu_{usual}, \sigma_{usual}^2\right)$ otherwise. In the same way the value of $\sigma_{cont(k)}$ is generated from a normal distribution with parameters $\mathcal{N}\left((0.5 \cdot \mu_{dense}), (0.25 \cdot \mu_{dense})^2\right)$ if the individual's coordinates M_k belong to any area of high density P_j for some $j = 1, 2, \ldots \mathcal{J}$, and from a normal distribution with parameters $\mathcal{N}\left((0.5 \cdot \mu_{usual}), (0.25 \cdot \mu_{usual})^2\right)$ otherwise. The corresponding means μ_{dense} and μ_{usual} are multiplied by 0.7 an 0.5 for individuals who are younger than 10 and older than 60 years due to the assumed limited social mobility for those age groups. The resulting values are rounded to integers after they are generated.

- The parameter $p_{cont(k)}$ for individual $\tilde{Q}_k$ defines the probability of any infection-induced contacts for a single day when the quarantine measures are not implemented. The values of $p_{cont(k)}$ are generated once for each individual $\tilde{Q}_k$ from a log-normal distribution ($\mathcal{LN}$) with parameters $\left(\mu_{ocp}, \sigma_{ocp}^2\right)$. The log-normal random variable X with parameters $\left(\mu_{ocp}, \sigma_{ocp}^2\right)$ is generated as $X = \exp\left(\sigma_{ocp} Z + \mu_{ocp}\right)$ where Z is generated from the standard normal distribution (i.e. $Z \sim \mathcal{N}(0,1)$). The detailed formulas for the μ_{ocp} and σ_{ocp}^2 in terms of mean and variance of the distribution X are described earlier in (7). The abbreviation ocp in the subscript stands for the "ordinary contact probability". The parameter μ_{ocp} is equal to the population-level parameter denoted as $Ordinary\_Contact\_Probability$ which is fitted for the model. The value σ_{ocp} is assumed to be small and fixed at 0.05. The values of the μ_{ocp} are multiplied by 0.8 and 0.6 for individuals who are younger than 10 and older than 60 years due to the assumed limited social mobility for those age groups. The generated probability values $p_{cont(k)}$ for each $\tilde{Q}_k$ are checked to be within $[0;1]$ range and re-generated if they are larger to ensure the correct boundaries for the probability values. The value of the $p_{cont(k)}$ is used for the binomial distribution with a single trial to a t every time step t to generate the presence of absence of contacts at a given day i.e. from the distribution $\mathcal{BIN}\left(1, p_{cont(k)}\right)$.

- The individual's reproduction number $R_{0(k)}$ is saved from the simulations for every infected person and contains the number of the other individuals that this person infects during the simulations. The numbers $R_{0(k)}$ across individuals and simulation runs are used to compute the averaged values and the corresponding confidence bands that are used as estimates of the population basic reproduction number $\mathcal{R}_0$.

- The $sever_k$ parameter is generated as an outcome of a trinomial distribution with a single trial with probabilities $\mathcal{MN}\left(1, (p_{1(g)}, p_{2(g)}, p_{3(g)})\right)$ which is a special case of a multinomial distribution. The first probability subscript contains the index of the disease severities described for (1). The second subscript g emphasizes that those probabilities are specific to age groups. There are three age groups within the model which are defined as:

$$G = \Big\{(< 20), (20 \text{ - } 50), (> 50)\Big\}. \tag{11}$$

- The duration of treatment parameter dur_k is generated from the normal distribution $\mathcal{N}\left(\mu_{dur(sever)}, \sigma^2_{dur(sever)}\right)$, which depends on disease severity. The resulting duration is rounded to integer values to be represented in days. The generated values that are smaller than 3 are changed to 3 since three days are assumed to be the shortest disease duration period [3] [4] [5].

- The status $(st_k(t))$ of the individual $\tilde{Q}_k$ is defined as $st_k(t) = 0$ when t is the reporting date for that individual and is updated for the other time steps t within the simulation. The initially reported individuals $\tilde{Q}_k$ have the status $st_k(t) = 1$ before the reporting data which changes to $st_k(t) = 0$ on the day of reporting.

The entire set of originally reported cases that corresponds to the reporting times $\tilde{t}_1, \tilde{t}_2, \ldots, \tilde{t}_{\tilde{\mathcal{S}}}$ is denoted as $\boldsymbol{D}$ and is presented with the global indexing in (4). The same set $\boldsymbol{D}$ can also be presented as a union of sets $\boldsymbol{D}_i(t)$ defined in (6) across all local epicenter $i = 1, 2, \ldots, \mathcal{I}$ at all time points up to and including the baseline time $t = 0$ and has the following representation:

$$\boldsymbol{D} = \bigcup_{t=-\tilde{\mathcal{S}}}^{0} \bigcup_{i=1}^{\mathcal{I}} \boldsymbol{D}_i(t). \tag{12}$$

In the formula (12) the number of reported cases within each epicenter $\boldsymbol{D}_i(t)$ during time t is different from epicenter to epicenter and is equal to $\Delta n_i(t)$. In the simulation every reported individual $\tilde{Q}_k$ from $\boldsymbol{D}$ is infectious with the status $st_k(t) = 1$ during the time interval $[tinf_k; tinf_k + det_k]$ and is isolated and is no longer able to infect the others after the time $tinf_k + det_k$ with the status $st_k(t) = 2$. The actual simulation starting time that accounts for all infectivity periods that precede the individual's reporting is denoted as T_{min} and is defined as:

$$T_{min} = \min_{\tilde{Q}_k \in \boldsymbol{D}} tinf_k. \tag{13}$$

Generation of the New Infections Within the Model

Every individual Q_k within the model is represented by the class (1). During the time indexes from interval $t \in T_{inf(k)} = [tinf_k; tinf_k + det_k]$ the individual Q_k has the status $st_k(t) = 1$ and can transmit infection to the others. Therefore, at each time step $t \in T_{inf(k)}$ the individual Q_k infects $inf_k(t)$ new individuals with probability $p_{cont(k)}$

and parameters $cont_k$. The lists of individuals infected by Q_k during the time slot t are denoted as $\boldsymbol{L}_k(t)$ and have the format:

$$\boldsymbol{L}_k(t) = \left\{I_1, I_2, \ldots, I_{inf_k(t)}\right\}, \tag{14}$$

where the total number of those newly infected individuals $inf_k(t)$ in the list $\boldsymbol{L}_k(t)$ depends on both on the infecting individual k and on the time slot t. The individuals within the list (14) have identical structure to the class described in (1) and have the format:

$$I_l = \Big((x_l, y_l), tinf_l, det_l, stg_l, age_l, rad, p_{cont(l)}, cont_l, R_{0(l)}, sever_l, dur_l, st_l(t)\Big) \tag{15}$$

where $l = 1, 2, \ldots, inf_k(t)$. The procedures for the $\boldsymbol{L}_k(t)$ simulation is outlined as follows:

- The amount of infected contacts $inf_k(t)$ for every individual $\tilde{Q}_k$ for each day t is generated based on the set of rules and assumptions outlined in details in the definition of $inf_k(t)$ for classes (1).

- For every infected contact index $l = 1, 2, \ldots, inf_k(t)$ the individual class (15) is simulated and denoted as I_l. The new individuals defined by (15) are simulated in the same way as the reported cases $\tilde{Q}_k$ that are introduced into the model in the beginning. The only difference is the simulated locations of the new individuals which are generated in relation to the new center $M_k(x_k, y_k)$ defined by $\tilde{Q}_k$ rather than the center C_i of some epicenter E_i for $i \in \{1, 2, \ldots, \mathcal{I}\}$ to which the individual $\tilde{Q}_k$ belongs to.

- The parameter $p_{cont(l)}$ for individual I_l defines the probability of any infection-induced contacts for each day when the quarantine measures are or are not implemented. In the first case the values $p_{cont(l)}$ are generated from a log-normal distribution $(\mathcal{LN})$ with parameters $\left(\mu_{ocp}, \sigma^2_{ocp}\right)$ in the same was as for the index cases $\tilde{Q}_k$ and the results are rounded to integer values. The subscript *ocp* stands for the *Ordinary_Contact_Probability* parameter. In the second case the values $p_{cont(j)}$ are generated from a log-normal distribution $(\mathcal{LN})$ with parameters $\left(\mu_{icp}, \sigma^2_{icp}\right)$ and the results are rounded to integer values. The subscript *icp* stands for the *Isolated_Contact_Probability* parameter. The shift from the *Ordinary_Contact_Probability* to the *Isolated_Contact_Probability* parameter is controlled by the *Quarantine_In* time point. The shift in the distribution of parameters for $p_{cont(l)}$ from the *ocp* to *icp* set happens at time index *Quarantine_In* i.e. when $t > Quarantine\_In$.

- For the newly simulated infected individuals I_l for all $l = 1, 2, \ldots, inf_k(t)$ the infection time is assigned to t i.e. $tinf_l = t$ and the status changes at time $t + 1$ i.e. $st_l(t + 1) = 1$.

The simulation of infected individuals defined in (15) is implemented sequentially for every time slot $t \in [T_{min}; T_{max}]$ and the modeled individuals at time t are combined in the overall list $\boldsymbol{\mathcal{L}}(t)$. The overall list $\boldsymbol{\mathcal{L}}(t)$ contains all of the detected individuals $\tilde{Q}_k$ across all local epicenters $i = 1, 2, \ldots \mathcal{I}$ that are incorporated into the model from the data as well as the lists of newly infected individuals $\boldsymbol{L}_k(t)$ that appear during the simulation process steps. For the smallest times slot $t = T_{min}$ it is assumed that $\boldsymbol{\mathcal{L}}(t) = \boldsymbol{\mathcal{L}}(T_{min})$ contains only the earliest model input cases from the list $\boldsymbol{D}(t) = \boldsymbol{D}(T_{min})$. The overall lists for subsequent time slots $\boldsymbol{\mathcal{L}}(t + 1)$ are formed sequentially from the lists $\boldsymbol{\mathcal{L}}(t)$ for $t \in [T_{min}; (T_{max} - 1)]$ via the following two scenarios:

- The first scenario corresponds to the time slots $t \leq 0$ i.e. before the selected baseline simulation time. In this case both the reported individuals $\boldsymbol{D}(t)$ and the lists $\boldsymbol{L}_k(t)$ of individuals infected during time t by all infectious individual indexed by all k-s are incorporated into the simulation for the next time slot $t+1$.
  - If the time index $t \leq 0$ i.e. before the selected baseline simulation time then the newly generated list $\boldsymbol{\mathcal{L}}(t+1)$ is initially formed from the previous list $\boldsymbol{\mathcal{L}}(t)$ and expanded by adding the detected individuals from the reported data that correspond to time $\boldsymbol{D}(t+1)$ across all epicenter i.e. with the list

$$\boldsymbol{D}(t+1) = \bigcup_{i=1}^{\mathcal{I}} \boldsymbol{D}_i(t+1) \tag{16}$$

    where the number of individuals in each epicenter i is equal to $\Delta n_i(t+1)$.
  - All individuals within the list $\boldsymbol{\mathcal{L}}(t)$ that are indexed by k are checked for the infectiousness condition $t \in T_{inf(k)}$. If the condition holds then the list of individual $\boldsymbol{L}_k(t)$ that is infected by individual k at time t is added to the list $\boldsymbol{\mathcal{L}}(t+1)$.
  - If for the individual indexed by k from $\boldsymbol{\mathcal{L}}(t)$ and for the current time t the condition $tinf_k + det_k < t$ holds, the individual is still moved to the list $\boldsymbol{\mathcal{L}}(t+1)$. The individual's status, however, is changing to quarantined (i.e. $st_k(t+1) = 2$) and the given individual stops infecting the others.
  - If for the individual indexed by k from $\boldsymbol{\mathcal{L}}(t)$ and for the current time t the condition $tinf_k + det_k + dur_k < t$ holds, the individual is still moved to the list $\boldsymbol{\mathcal{L}}(t+1)$. The individual's status, however, is changing to either recovered (i.e. $st_k(t+1) = 3$) if the individual's severity variable $sever_k = 2$ or 3, or deceased (i.e. $st_k(t+1) = 4$) if the individuals severity variable $sever_k = 1$.
- The second scenario corresponds to the time slots $t > 0$ i.e. after the selected baseline simulation time. In this case only the lists $\boldsymbol{L}_k(t)$ of individuals infected during time t by the infectious individual with index k are incorporated into the simulation for the next time slot $t+1$.

The Overall Model Flow

The entire modeling process can be summarized via the following steps:

- The model input time interval is determined by fixing the first $\tilde{\mathcal{S}}$ reporting indexes out of the total $\mathcal{S}$ where $\tilde{\mathcal{S}} < \mathcal{S}$. Those indexes correspond to the reporting time slots $\tilde{t}_1, \tilde{t}_2, \ldots, \tilde{t}_{\tilde{\mathcal{S}}}$. This completely defines the list of reported index cases $\boldsymbol{D}$ from (4) that are used as the model initial conditions. The baseline time of the model $t = 0$ is assumed to correspond to $\tilde{t}_{\tilde{\mathcal{S}}}$.
- The individuals from the reported set $\boldsymbol{D}$ that are defined in (4) are assigned to the local epicenters of the future epidemic E_i for $i = 1, 2, \ldots, \mathcal{I}$ based on the available (from the input data) geographic distribution.
- The geographic data about the areas of high density P_j for $j = 1, 2, \ldots, \mathcal{J}$ are incorporated into the model.
- The model is initialized with the index cases from $\boldsymbol{D}$. Based on those index cases that are defined in (4) the initial infection time T_{min} is determined. This step is necessary to incorporate the infection times that have been present before the first reporting time $\tilde{t}_1$ into the model.

- The final time point of the stochastic simulations T_{max} is defined by the user based on the desired study and prediction goals.
- The initial list of infected individuals $\boldsymbol{\mathcal{L}}(T_{min})$ is initialized at time T_{min} only with the earliest model input cases from the list $\boldsymbol{D}(T_{min})$.
- The infected list of individuals $\boldsymbol{\mathcal{L}}(t+1)$ for the time slot $t+1$ is generated sequentially for all $t \in [T_{min}; T_{max}-1]$ based on the list of individuals $\boldsymbol{\mathcal{L}}(t)$ from previous time slot t as described in the new infection generation process and the individual's characteristics within the list $\boldsymbol{\mathcal{L}}(t+1)$ are updated at this time step $t+1$.

Based on the lists $\boldsymbol{\mathcal{L}}(t)$ at every time slot $t \in [T_{min}; T_{max}]$ the infected modeled population summaries can be computed and summarized. In particular, the total number of currently infected but not identified individuals (i.e. those with the status $st(t) = 1$) is saved into $Inf(t)$ variable for every t. The total number of treated or quarantined individuals (i.e. with the status $st(t) = 2$) is saved into $Treat(t)$ variable for every t. The total number of recovered individuals (i.e. with the status $st(t) = 3$) is saved into $Recov(t)$ variable for every t. The total number of deceased individuals (i.e. with the status $st(t) = 4$) is saved into $Dead(t)$ variable for every t. Those numbers are used in the model calibration procedures, epidemiological summaries and in the model predictions. The model input utilizes only the first $\tilde{\mathcal{S}}$ reported indexes with the corresponding reported times $\tilde{t}_s$ for $s = 1, 2, \dots, \tilde{\mathcal{S}}$ with the total number of reported indexes equal to $\mathcal{S}$ and $\tilde{\mathcal{S}} < \mathcal{S}$. The remaining reported indexes $\tilde{\mathcal{S}} + \tau, \tilde{\mathcal{S}} + \tau + 1, \dots, \mathcal{S}$ for some integer $\tau \geq 0$ are divided into the two groups:

$$\left\{\tilde{\mathcal{S}} + \tau, \tilde{\mathcal{S}} + \tau + 1, \dots, \dot{\mathcal{S}}\right\} \text{ and } \left\{\dot{\mathcal{S}} + 1, \dot{\mathcal{S}} + 2, \dots, \mathcal{S}\right\}. \quad (17)$$

The first group of the reported indexes from (17) is used for the model calibration and estimation of the unknown parameters. The second group of the reported indexes from (17) is used to evaluate the quality of the model predictions. The Massachusetts surveillance data that are used for the model calibration, validation and predictions are freely available at the Massachusetts Department of Public Health web site [6]. The first reported date which corresponds to the time index $\tilde{t}_1$ in the model is March 13, 2020. The latest reported date that is used for the model input is March 26, 2020 which corresponds to the time index $\tilde{t}_{\tilde{\mathcal{S}}}$ in the model. The time indexes that correspond to $\tilde{t}_{\tilde{\mathcal{S}}+\tau}$ and $\tilde{t}_{\dot{\mathcal{S}}}$ are April 14, 2020 and April 22, 2020 respectively. The parameters optimization is performed by minimizing the sum of squared differences between the model-produced outputs and the calibration data by using the Nelder–Mead numerical minimization method [7].

The Model Calibration Details

The set of global parameters denoted as $\boldsymbol{HP}$ that the model is optimized over is presented below:

$$\boldsymbol{HP} = \left\{Detect, Ordinary\_Contact\_Prob, Isolated\_Contact\_Prob, Usual\_cont, Dense\_cont\right\}.$$

Based on the model-produced lists $\boldsymbol{\mathcal{L}}(t)$ at every time slot t the model-produced summaries of the generated infected population can be computed and summarized for a given set of parameters from $\boldsymbol{HP}$ which includes the time series of $Inf(t)$, $Treat(t)$, $Recov(t)$ and $Dead(t)$. In the proposed model formulation the set of global parameters $\boldsymbol{HP}$ is estimated based on the $Total(t) = Treat(t) + Recov(t) + Dead(t)$ information about the infected individuals (i.e. those with the statuses $st(t) = 2, 3, 4$) time series produced by the model for time slot $t \in [T_{init}; T_{term}]$. The time slots between T_{init} and

T_{term} correspond to the calibration period from (17) and therefore $T_{init} = \tilde{t}_{\hat{\mathcal{S}}+\tau}$ and $T_{term} = \tilde{t}_{\hat{\mathcal{S}}}$ in notations from (17). The reported data values [6] are summarized into the the data-produced time series $TotalAct(t)$ about the infected and detected individuals for the time slots $t \in [T_{init}, T_{term}]$.

The objective function $ObjFunc\,(\boldsymbol{HP})$ for minimization across the set of parameters from $\boldsymbol{HP}$ is the standardized sum of squared differences between the model produced outputs and the reported data time series:

$$ObjFunc\,(\boldsymbol{HP}) = \sqrt{\frac{1}{[T_{term} - T_{init}]} \sum_{t=T_{init}}^{T_{term}} \Big[Total(t) - TotalAct(t)\Big]^2}. \quad (18)$$

The Nelder–Mead [7] numerical minimization method for multiple dimensions has been used to minimize $ObjFunc\,(\boldsymbol{HP})$ function from (18) with respect to $\boldsymbol{HP}$. The initial values for the algorithm $\boldsymbol{HP}_0$ are selected from the plausible ranges of parameters of interest which provide a good visual alignment between the collected data and the model produced output.

The model availability

The model has been implemented in multiple environments which include the application tool for Microsoft Windows [8] and the web prediction tool [9] [10] (summaries only). The model application tool for Microsoft Windows is freely available under the terms of the MIT license [11]. The model parameters can either be changed manually by the user within the parameters table that is used by the tool or, more conveniently, within the tool graphic user interface (GUI). The example of the parameter's table in presented in Fig. A and the tool source code, the application, and the relevant tool documentation are available on GitHub [8].

The current model tool has been calibrated based on the state of Massachusetts (United States) incidence data [6]. In addition to that the user has an option to adjust interactively the tool parameters which include, in particular, the transmission parameters and the quarantine implementation dates(s). Overall, the proposed framework and the code are fairly general and can be adopted for other areas and territories where the demographics of the incidence cases and population characteristics are known with at least some geographic precision, and where the rapid evaluations of social distancing measures have to be quantified.

```
InitContProb=0.192
IsolContProb=0.127
ShowSimulProgr=1
AutoSize=1
ShowEpicenters=0
LeapYear=1
LogNormal=1
NormalDistrAge=0
DrawInfect=1
RedR0Hist=1
R0DistrFunc=1
TimeIsol=11
MaxSimulTime=111
DetDay=13
InitOpt=19
TermOpt=27
ForeDay=52
ForeIter=250
SimType=0
WeekIter=100
WeekCount=6
ContCirclesCount=6
ContCirc_0=729,215,57,
ContCirc_1=746,310,57,
ContCirc_2=797,428,51,
ContCirc_3=552,237,47,
ContCirc_4=300,303,50,
ContCirc_5=162,265,47,
HiperParamCount=5
HiperParam_0=3.55,1.30
HiperParam_1=50.00,20.00
HiperParam_2=1000.00,250.00
HiperParam_3=2.36,0.50
HiperParam_4=3.23,1.20
DiseaseForm_0= 3.1, 6.9,10.4
DiseaseForm_1= 3.5,10.4,15.2
DiseaseForm_2=93.4,82.7,74.4
DiseaseDuration_0= 8.7, 3.3
DiseaseDuration_1=11.8, 3.3
DiseaseDuration_2= 7.8, 4.7
```

Fig A. The illustration of the tool configuration file. The configuration file parameter values are loaded by the tool when the tool is initially launched. Then the user has an option to use the loaded parameter's values, to adjusted them manually or to use the tool estimation procedures.

References

1. Milligan GN, Barrett AD. An Essential Guide. Wiley Online Library; 2015.

2. worldpopulationreview.com;. https://worldpopulationreview.com/states/massachusetts-population/.

3. Chen J, Qi T, Liu L, Ling Y, Qian Z, Li T, et al. Clinical progression of patients with COVID-19 in Shanghai, China. Journal of Infection. 2020;.

4. McMichael TM, Currie DW, Clark S, Pogosjans S, Kay M, Schwartz NG, et al. Epidemiology of Covid-19 in a long-term care facility in King County, Washington. New England Journal of Medicine. 2020;.

5. Lauer SA, Grantz KH, Bi Q, Jones FK, Zheng Q, Meredith HR, et al. The incubation period of coronavirus disease 2019 (COVID-19) from publicly reported confirmed cases: estimation and application. Ann Intern Med. 2020;10:M20–0504.

6. www.mass.gov;. https://www.mass.gov/info-details/archive-of-covid-19-cases-in-massachusetts.

7. Nelder JA, Mead R. A simplex method for function minimization. The computer journal. 1965;7(4):308–313.

8. github.com;. https://github.com/quantori/COVID19-MA-Transmission.

9. tableau.com;. https://public.tableau.com/profile/quantori#!/vizhome/ModelingCovidMassachusets/COVID-19Massachusettsforecast.

10. tableau.com;. https://public.tableau.com/profile/quantori#!/vizhome/ModelingCovidMassachusets_Map/InteractiveMap.

11. MIT Licence;. https://opensource.org/licenses/MIT.
