# Supplementary material for: Development of an interactive, agent-based local stochastic model of COVID-19 transmission and evaluation of mitigation strategies illustrated for the state of Massachusetts, USA
Source: PLoS One. 2021 Feb 17;16(2):e0247182. doi: 10.1371/journal.pone.0247182 (PMC7888623; doi:10.1371/journal.pone.0247182)
Supplement: Attachment — Submitted filename: Responses_to_reviewers_VK_AK_Cleaned_FINAL.pdf [file pone.0247182.s002.pdf]

Reviewer #1: This study developed and applied an agent-based for MA to assist local decision-makers about interventions related for the COVID-19 pandemic. There were several unique features of the study such as de-identification of reported case data but overall the parameterization and epidemiological soundness of the model structure are lacking. The authors are to be commended on their effort to make the data and code available as open source.

Lines 48-54: It is not clear what is meant by "localized".

We meant the model that can be applied to local community settings to help local public health officials to act accordingly in their area of responsibility. We changed the wording to reflect that.

Line 83: What exactly is meant by "infection acquisition"? Is it asymptomatic or symptomatic? Are you referring to disease transmission? This is not clear.

We meant either infection (symptomatic or asymptomatic). We clarified that in the text.

Line 87: duration of disease infections does not equate with disease severity. I suggest the author gain a better understanding of infectious disease epidemiology.

We do understand that. Here we meant something else. We meant to say that individual has different disease stages that are individual-specific within the model and those stages characterize the infectivity of each specific individual at each state. This is described in details in the Supplement. We changed the wording both in the main body and in the supplement to clarify that.

Line 94: I am disturbed by the statement that cont_k is randomly generated each day. What social/biological process is assumed to justify randomness?

Here we present a fairly general framework. The number of newly infected individuals by individual k is generated randomly from a distribution whose parameters depend on the disease stages of the individual k. In the model it is assumed that individual's infectivity depends on the disease stages and is individual-specific which justifies randomness. The details of the generation process have been described in the Supplement (page 6).

Line 104: Once again, it is not clear why a random value is being used to determine disease severity? These parameters should be informed by the published literature.

The goal was to present a framework. The distribution of the disease severity sever_k parameter is generated from a trinomial distribution. The parametrization of this distribution can be done based on the known proportions of the severe, mild and lethal cases for the area where the model is applied. The available in literature parameters are different from location to location and from outbreak to outbreak. The set of parameters in the model allowed us to calibrate the model.

Line 113-119: If an individual is detected as a reported case, then is it not true that they are infected? How else would they have been detected if they were not infected? Supposedly in MA cases are reported after testing positive for SARS-CoV-2. If this is not the case, then more details are needed on what is meant by "reported" and how this is not the same as being "infected" (symptomatic or asymptomatic).

The detected individuals (stk(t) = 0) here are referred to the reported data and they are imputed into the model at the early time stages. The model uses reported patients as an input. The other individuals infected within the model based on the detected and newly generated cases receive the remaining statuses. The detected individuals within the model are assumed to be immediately quarantined (isolated) and they cannot infect the others.

Line 130: An epicenter means a high number of localized infections and does not have anything to do with a radius. I am not sure how this parameterization of an epicenter is grounded in theory.

The model is a geospatial approximation which is motivated by the resolution of the available data. The available data are the lattice data where individuals are reported for each county. The epicenter of each county is defined by the geographic center of that county and the corresponding radius in defined by the size of the corresponding county.

Table 1 and 2: It would be helpful to report these as attack rates rather than total number of cases. Also, if you can break these down by age groups, then that would be another type of model calibration.

This functionality is not in the model right now but we are planning to extend and update our model in the future. Therefore we have provided the permanent repository link on GitHub for newer versions of the code.

The first three pages of the appendix is a repetition of the methods section.

We did that intentionally. We wanted to keep the flow of the detailed model parameterization in the appendix. We can remove it if requested but we believe it will be easier for the reader in this way.

Model parameters and data sources should be presented as a table for ease of following.

Data have been included as a table in the source code in the epicenters table. The table stored online contains all the information about the epicenters and can be adjusted by the reader if needed. We kept it online intentionally since they can be updated in the future if more relevant data appear.

No details are given on how mu_det and sigma_det were estimated in the model.

Those are hyper parameters which initial values are defined by the user in the way that the model output roughly matches the available data. Those parameters are optimized further by the algorithm as it is described in the supplement.

No justification is provided for choice of parameters or formulas used to describe length of disease stages.

The initial values before the optimization are defined in the way that they 1) are biologically plausible, 2) the model outputs roughly match the available data. The parameters are optimized further as described in the supplement.

No justification given for several other disease model parameters (e.g., social contacts)

The initial values before the optimization are defined in the way that they 1) are biologically plausible, 2) the model outputs roughly match the available data. The parameters are optimized further as described in the supplement.

Reviewer #2: Reproducibility report has been uploaded as an attachment.

The copy of the report is included.

Reviewer #3: The manuscript describes an interactive agent-based model designed to evaluate the impact of non-pharmaceutical interventions to mitigate the impact of COVID-19 in Massachusetts, USA. Given the ability of agent-based models to represent individual characteristics, the authors argue that this type of model has the potential to be more appropriate for certain transmission scenarios that depend on local demographic and geographic variables. The aim of the study is to provide a tool to decision makers to plan for the timing of quarantines and their reopening strategies. The model was initialized with epicenters based on surveillance epidemiological data and model parameters were calibrated to represent the total information of infected individuals (treat, recovered, and dead). Qualitatively, the model matches the cumulative number of cases and deaths. Finally, the authors evaluated a set of three different starting times for lock-down strategies in which individuals reduce their contact rates.

The model is interesting and addresses an important public health issue. However, from the manuscript, I was unable to see the particular public health impact or advance in computational biology of this study. I describe my concerns below.

1. Lack of clarity on relevance of this study and analysis of the results. The authors described their model but the analysis was very limited. Only three hypothetical scenarios of quarantine were analyzed as a proof of concept. Also, several parameters are listed in the model, and the list of calibrated parameters are listed in the supplementary material. However, I did not find any information on the numerical values for any of the parameters. Furthermore, the description of the calibration step does not specify how the stochasticity of the model was accounted for the nelder-mead optimization algorithm. Finally, the authors

used cumulative cases and deaths to adjust the model parameters. Using incidence instead of cumulative numbers could improve the fit of the model parameters.

The goal of this manuscript was to present a locally-applicable framework that can be used by local officials to investigate the epidemic behavior over a period of time to plan the public health intervention. For the model that utilizes epidemiological data the outputs that can be compared with reported cases and death are used for model fitting. Otherwise such models have to rely on modeling assumptions. The fits were performed to the distribution parameters of the output model which accounted for stochasticity.

2. The introduction and discussion sections of the manuscript lacked a comparison to other models as well as a description of the limitations of the model. Many other modeling tools are currently available study the impact of interventions on COVID-19. For instance, Aleta et al. used an agent-based model to evaluate the impact of social distancing, contact tracing, and quarantine in a second wave of the COVID-19 epidemic in Boston (https://www.mobs-lab.org/uploads/6/7/8/7/6787877/tracing_main_may4.pdf). Putting the proposed model in the context of other available models could help to understand the limitations and unique features of this model. This comparison can also help to understand the relevance of this study.

Since the initial manuscript submission more agent-based modeling papers have appeared. We have updated the literature review and discussion. We have also updated the list of published papers and the discussion section.

Other minor comments:

- "Predicts" is a strong statement, how was it tested?

The model was fitted in multiple steps. The input data corresponded to the early stages of the epidemic, which was followed by the calibration data period. The calibration data period followed the input data period and was used to calibrate the model. By predictions here we meant the model-produced output beyond the calibration period. The predicted model output was aligned and compared with the actual cases that were available beyond the calibration period in time.

- Lines 17-18. Please define terms of quarantine and social distancing.

Done. We have provided the description.

- Lines 48-49. What do the authors mean with successfully applied to study respiratory diseases?

Here we meant that the model FluTE that is designed for influenza has been successfully applied for other respiratory infections that include SARS-CoV-2. We updated the initial goals of FluTE model in the manuscript.

- Are there any other models other than FluTE?

Since the initial manuscript submission more agent-based modeling papers have appeared. We have updated the literature review and discussion. We have also updated the list of published papers and the discussion section.

- How are the authors incorporating latent and incubation periods?

In the current model implementation there are three infection periods. The latent and incubation period are blended into the first period. The details are provided in the Supplement.

- What's the difference between asymptomatic and symptomatic infections, are they all the same?

The asymptomatic infections are included into the first period of the disease. If the entire course of the disease is asymptomatic in will not be registered in the model.

- What's the probability of symptoms by age?

The age parameter is used to define the distribution of the disease severities. In this case the disease severity is affected by age. This mechanism is described in the Supplement.

- Are all the individuals equally susceptible?

We are generating the newly infected individuals with certain probabilities that is affected by multiple characteristics that are described in the supplement such as age group and location.

- Line 132: There should be a reference for the surveillance epidemiological data that the authors are using.

Thank you for pointing it out. We have added the reference.

- Line 219: How did the authors deal with the stochasticity in the calibration step?

The fits were performed to the distribution parameters of the output model which accounted for stochasticity.

- The uncertainty and the median values of the model are surprisingly smooth for a stochastic model. Are the authors reporting the daily or weekly numbers?

he predictions and the uncertainty bands were taken pointwise for each day and the percentiles were taken across 500 replications. If the reader runs the same code with a smaller number of replications (5, 10, 50 etc) it will be possible to see less smooth time.

- By definition, R0 only makes sense at the beginning of the simulation with all susceptible population. The authors present figure 2 with pre-quarantine and post quarantine R0. I think they refer to R(t)

The basic reproduction number is typically defined for compartmental models and is derived from the set of parameters. Here we had an option to estimate the average number of infections caused by the infected individuals from the infected population. Since those numbers are expected to be different before and after the interventions we have provided both numbers.
