# Supplementary material for: Development of an interactive, agent-based local stochastic model of COVID-19 transmission and evaluation of mitigation strategies illustrated for the state of Massachusetts, USA
Source: PLoS One. 2021 Feb 17;16(2):e0247182. doi: 10.1371/journal.pone.0247182 (PMC7888623; doi:10.1371/journal.pone.0247182)
Supplement: Attachment — Submitted filename: Response_CLEAN.pdf [file pone.0247182.s003.pdf]

2.1. Please provide an amended Funding Statement declaring this commercial affiliation, as well as a statement regarding the Role of Funders in your study. If the funding organization did not play a role in the study design, data collection and analysis, decision to publish, or preparation of the manuscript and only provided financial support in the form of authors' salaries and/or research materials, please review your statements relating to the author contributions, and ensure you have specifically and accurately indicated the role(s) that these authors had in your study. You can update author roles in the Author Contributions section of the online submission form.

Please also include the following statement within your amended Funding Statement.

“The funder provided support in the form of salaries for authors [insert relevant initials], but did not have any additional role in the study design, data collection and analysis, decision to publish, or preparation of the manuscript. The specific roles of these authors are articulated in the ‘author contributions’ section.”

If your commercial affiliation did play a role in your study, please state and explain this role within your updated Funding Statement.

Funding Statement (also duplicated in the submission Cover Letter):

The authors Vladimir Koniukhovskii (VK), Vladimir Shvartc (VS) and Konstantin Barsukov (KB) are employed by the commercial company EPAM Systems in Saint Petersburg, Russia.

The authors Yuriy Gankin (YG) and Semyon Semyonov (SS) are employed by the commercial company Quantori in Cambridge, Massachusetts, United States.

EPAM Systems provided support in the form of salaries for authors VK and VS, but did not have any additional role in the study design, data collection and analysis, decision to publish, or preparation of the manuscript. The specific roles of these authors are articulated in the ‘author contributions’ section. The authors worked on the publication at their leisure time unrelated to their job duties.

Quantori provided support in the form of salaries for authors YG and SS and relevant publication fees. YG is a Chief Scientific Officer (CFO) of Quantori was an inspirer for the project due to COVID-19 situation and SS worked on the project under the supervision of YG. The specific roles of these authors are articulated in the ‘author contributions’ section.

Alexander Kirpich (AK), Pavel Skums (PS), Thomas A. Weppelmann (TAW), and Evgeny Imyanitov (EI) received no funding for their work on the project. The specific roles of these authors are articulated in the ‘author contributions’ section.

2.2. Please also provide an updated Competing Interests Statement declaring this commercial affiliation along with any other relevant declarations relating to employment, consultancy, patents, products in development, or marketed products, etc.

Within your Competing Interests Statement, please confirm that this commercial affiliation does not alter your adherence to all PLOS ONE policies on sharing data and materials by including the following statement: "This does not alter our adherence to PLOS ONE policies on sharing data

and materials.” (as detailed online in our guide for authors http://journals.plos.org/plosone/s/competing-interests) . If this adherence statement is not accurate and there are restrictions on sharing of data and/or materials, please state these. Please note that we cannot proceed with consideration of your article until this information has been declared.

https://journals.plos.org/plosone/s/competing-interests

Competing Interests Statement (also duplicated in the submission Cover Letter):

The authors have declared that no competing interests exist. Alexander Kirpich (AK), Pavel Skums (PS), Thomas A. Weppelmann (TAW), and Evgeny Imyanitov (EI) received no funding for the work on the project. The authors Vladimir Koniukhovskii (VK), Vladimir Shvartc (VS) and Konstantin Barsukov (KB) are employed by the commercial company EPAM Systems in Saint Petersburg, Russia. The authors Yuriy Gankin (YG) and Semyon Semyonov (SS) are employed by the commercial company Quantori in Cambridge, Massachusetts, United States.

The commercial affiliations of authors VK, VS, KB, YG, and SS do not alter their adherence to PLOS ONE policies on sharing data and materials. The data and the source code are made publicly available which is outlined in the manuscript. Quantori provided support in the form of salaries for authors YG and SS and relevant publication fees. YG is a Chief Scientific Officer (CFO) of Quantori was an inspirer for the project due to COVID-19 situation and desire to contribute. This will serve the overall good and is aligned with Quantori’s values, policies and goals.

Please include both an updated Funding Statement and Competing Interests Statement in your cover letter. We will change the online submission form on your behalf.

Please know it is PLOS ONE policy for corresponding authors to declare, on behalf of all authors, all potential competing interests for the purposes of transparency. PLOS defines a competing interest as anything that interferes with, or could reasonably be perceived as interfering with, the full and objective presentation, peer review, editorial decision-making, or publication of research or non-research articles submitted to one of the journals. Competing interests can be financial or non-financial, professional, or personal. Competing interests can arise in relationship to an organization or another person. Please follow this link to our website for more details on competing interests: http://journals.plos.org/plosone/s/competing-interests

3. We note that Figure 1 in your submission contain map images which may be copyrighted. All PLOS content is published under the Creative Commons Attribution License (CC BY 4.0), which means that the manuscript, images, and Supporting Information files will be freely available online, and any third party is permitted to access, download, copy, distribute, and use these materials in any way, even commercially, with proper attribution. For these reasons, we cannot publish previously copyrighted maps or satellite images created using proprietary data, such as Google software (Google Maps, Street View, and Earth). For more information, see our copyright guidelines: http://journals.plos.org/plosone/s/licenses-and-copyright.

We require you to either (1) present written permission from the copyright holder to publish these figures specifically under the CC BY 4.0 license, or (2) remove the figures from your submission:

3.1. You may seek permission from the original copyright holder of Figure 1 to publish the content specifically under the CC BY 4.0 license.

We recommend that you contact the original copyright holder with the Content Permission Form (http://journals.plos.org/plosone/s/file?id=7c09/content-permission-form.pdf) and the following text:

“I request permission for the open-access journal PLOS ONE to publish XXX under the Creative Commons Attribution License (CCAL) CC BY 4.0 (http://creativecommons.org/licenses/by/4.0/). Please be aware that this license allows unrestricted use and distribution, even commercially, by third parties. Please reply and provide explicit written permission to publish XXX under a CC BY license and complete the attached form.”

Please upload the completed Content Permission Form or other proof of granted permissions as an "Other" file with your submission.

In the figure caption of the copyrighted figure, please include the following text: “Reprinted from [ref] under a CC BY license, with permission from [name of publisher], original copyright [original copyright year].”

3.2. If you are unable to obtain permission from the original copyright holder to publish these figures under the CC BY 4.0 license or if the copyright holder’s requirements are incompatible with the CC BY 4.0 license, please either i) remove the figure or ii) supply a replacement figure that complies with the CC BY 4.0 license. Please check copyright information on all replacement figures and update the figure caption with source information. If applicable, please specify in the figure caption text when a figure is similar but not identical to the original image and is therefore for illustrative purposes only.

The following resources for replacing copyrighted map figures may be helpful:

USGS National Map Viewer (public domain): http://viewer.nationalmap.gov/viewer/

The Gateway to Astronaut Photography of Earth (public domain): http://eol.jsc.nasa.gov/sseop/clickmap/

Maps at the CIA (public domain): https://www.cia.gov/library/publications/the-world-factbook/index.html and https://www.cia.gov/library/publications/cia-maps-publications/index.html

NASA Earth Observatory (public domain): http://earthobservatory.nasa.gov/

Landsat: http://landsat.visibleearth.nasa.gov/

USGS EROS (Earth Resources Observatory and Science (EROS) Center) (public domain): http://eros.usgs.gov/#

Natural Earth (public domain): http://www.naturalearthdata.com/

Thank you for pointing that out. We have changed the map in the tool and in Figure 1 to freely available map. The corresponding text have been added to the manuscript body to acknowledge the source.

The state of Massachusetts MassGIS data were used to produce the tool map. The data are available for download and are public records from the Bureau of Geographic Information (MassGIS), Commonwealth of Massachusetts, Executive Office of Technology and Security Services (ref provided).

[Note: HTML markup is below. Please do not edit.]

Reviewers' comments:

Reviewer's Responses to Questions

Comments to the Author

1. Is the manuscript technically sound, and do the data support the conclusions?

The manuscript must describe a technically sound piece of scientific research with data that supports the conclusions. Experiments must have been conducted rigorously, with appropriate controls, replication, and sample sizes. The conclusions must be drawn appropriately based on the data presented.

Reviewer #1: Partly

Reviewer #2: Yes

---

2. Has the statistical analysis been performed appropriately and rigorously?

Reviewer #1: Yes

Reviewer #2: Yes

---

3. Have the authors made all data underlying the findings in their manuscript fully available?

The PLOS Data policy requires authors to make all data underlying the findings described in their manuscript fully available without restriction, with rare exception (please refer to the Data Availability Statement in the manuscript PDF file). The data should be provided as part of the manuscript or its supporting information, or deposited to a public repository. For example, in addition to summary statistics, the data points behind means, medians and variance measures should be available. If there are restrictions on publicly sharing data—e.g. participant privacy or use of data from a third party—those must be specified.

Reviewer #1: Yes

Reviewer #2: Yes

4. Is the manuscript presented in an intelligible fashion and written in standard English?

PLOS ONE does not copyedit accepted manuscripts, so the language in submitted articles must be clear, correct, and unambiguous. Any typographical or grammatical errors should be corrected at revision, so please note any specific errors here.

Reviewer #1: Yes

Reviewer #2: Yes

5. Review Comments to the Author

Please use the space provided to explain your answers to the questions above. You may also include additional comments for the author, including concerns about dual publication, research ethics, or publication ethics. (Please upload your review as an attachment if it exceeds 20,000 characters)

Reviewer #1: Kirpich et al present an agent-based model of COVID spreading. They fit their model to data on COVID infections in Massachusetts. And provide an online tool to test their model.

There are a few major issues with the presentation and the results of the manuscripts:

1. The authors do not consider asymptomatic COVID patients in the model, but they could also spread the virus. So the fits provided only to symptomatic patients could be mis-parameterized due to the lack of the presence of asymptomatic patients in the model.

We agree that the question of asymptomatic infections is important and challenging. This happens due to the fact that asymptomatic individuals are unobserved and undetected. To improve the model computational tractability make the model better computationally tractable, we blended undetected asymptomatic and symptomatic infections into the same class called undetected (with the status st_k(t) =1 in lines 116-125 of the manuscript) since they all participate in transmission. The asymptomatic individuals within this class are incorporated via the three severity statuses (top of the page 7 form the supplement) that are generated for each individual. The first severity status is "mild" which incorporates asymptomatic as well. Then after an individual within this undetected class with any severity status is getting detected (i.e. tested and confirmed to have an infection) this individual is assigned the "detected" status (st_k(t) =1) and is immediately quarantined. This approach allows to incorporate all infected individuals with different disease severities into the infected status and to isolate the confirmed cases. The model parameters have been calibrated based on the detected cases and the output of the model presents the detected cases so the predicted ability for detected and reported cases within the model is preserved.

We also agree that this was not explained in the text explicitly enough, and we modified the description in the Methods section to emphasize that more and to make it clear. We have also provided the corresponding reference for the Supplement within the Methods section.

2. It is also a bit strange how virus spreading is handled. There are initial epicenters of the infections, but agents are not directly moving, rather just changing their contacts. The pandemic here spreads between places, but unclear how the spatial structure is taken into account – out of plotting the results on a map.

We believe that any spatial modeling of epidemics is challenging since the goal is the balance between the complexity of the parametrization and the ability to represent the transmission process. As a result the migration of individuals was not considered and we have focused on local transmissions for the specific geographic centers and regions. In the proposed model the generation of new contact's with certain probability and in certain randomly generated geographic proximity can serve as a substitute for moving since the process is intended to represent the transmission network.

At the same time in the proposed model we have incorporated the spatial structure in multiple ways. In particular, we have incorporated the local epicenters where disease is introduced that represent the real geographic locations. In addition to that high population density areas have been incorporated into out model to mimic real locations which affected the contact probabilities. The distance up to which a given selected individual can infect the other individuals is defined by rad_k parameter which is generated from a distribution which parameters are specific to mimic the population mobility. In addition to that the amount of contacts for a given individual on a given day is generated based on the distribution which accounts for high population density areas and which ultimately accounts for spatial distribution within the designated areas. Please refer to page 6 of the Supplement for more details.

We also agree that this process was not explained in the text explicitly enough, and we modified the description in the Methods section to emphasize the mechanism and to make it clear. We have also provided the corresponding reference for the Supplement within the Methods section.

3. Most of the details of the model are presented only in the supplement and even there some details are unclear. For instance, the authors discuss the effects of quarantines and social distancing, but it is not clear how the model is adjusted to take into account these effects. They mention 3 quarantine scenarios, but it seems these are just three separate times of quarantine initiation. Based on the text, only individuals with symptoms are quarantined, which is a far weaker restriction than what is generally applied by authorities.

We agree that quarantine measures can be implemented differently especially in complex models. In the model the quarantine measures are defined as follow: On a specific date the contact probability parameters change to reflect the reduction (but not the entire elimination) in contacts between the individuals. This mimics the real quarantine when social interactions and other activities and transmissions within the population are reduced but not eliminated completely, since the essential services (grocery stores, police, fire departments, hospitals, and warehouses) have to remain open. We have clarified in details the way the quarantine measures have been implemented in the Methods, the Model availability, and the Results sections of the manuscript to make it clear for the reads and to avoid possible confusions.

In the model terms it is represented as the shift in the distribution of parameters for pcont(l) from the ocp to icp after the time index Quarantine_In i.e. when t > Quarantine_In. This parameter and the quarantine start date are controlled and customized by the user. As an example of the model implementation three relevant quarantine scenarios have been presented in the manuscript. In particular, the first scenario corresponds to the quarantine date on March 29, 2020 i.e. the early reduction in contact probabilities and social distancing between individuals. The second scenario assumes the implementation of the quarantine measures on April 6, 2020, and the third scenario assumes the implementation of the quarantine measures on April 13, 2020. Those scenarios are compared in the Results section in terms of the number(s) of infected.

Initially, to save the manuscript space we have moved the details about the quarantine implementations to the page 7 of the supplement while kept the results within the main manuscript body. The user can also try deferent quarantine scenarios interactively within the tool. The instructions for that have been provided in the new ReadMe.pdf file that have been uploaded on GitHub. Upon request we have provided more details and additional clarifications in the manuscript about the quarantine measures implementations in the Methods, the Model availability, and the Results sections to avoid confusion. Those clarifications are expected to organically complement the supplement.

When the quarantine is implemented the contact probabilities are reduced for all individuals and not only for the symptomatic ones (supplemented page 7). This goes in addition to those individuals who are always individually isolate from further transmission (i.e. quarantined) after detection with the status st_k(t) = 2 which is assigned regardless of quarantine implementation.

4. In the responses to earlier referees it is stated that various answers and extensions were added to the text, but unclear how the text was eventually changed. No supporting file of tracked changes is provided. There is one tracked file for the supplement, but it seems only half a sentence was changed in that document.

Perhaps it was some misunderstanding. We have included 4 documents total in our previous submission. One was the main body document after all the changes which was embedded into the compiled submission PDF as pages. The other three (main document with tracked changes, supplement with changes and supplement with tracked changes) were included as links within the submission PDF that are duplicated below:

Massachusetts_Supplement_UPDATED.pdf

https://www.editorialmanager.com/pone/download.aspx?id=27130926&guid=028f9f1c-a626-46fa-bdfd-04fca9e3839c&scheme=1

Massachusetts_Supplement_UPDATED_TRACKED.pdf

https://www.editorialmanager.com/pone/download.aspx?id=27130928&guid=de1c0584-ae84-4634-9d02-23a356cb6f3f&scheme=1

Massachusetts_Draft_UPDATED_TRACKED.pdf

https://www.editorialmanager.com/pone/download.aspx?id=27130924&guid=0b8042aa-03fe-43c8-9413-89b7e071d1a3&scheme=1

We have tracked all the changes in both the main body and the supplement and they had been available for the review. We did make a small change to the main body but we changed a lot in the supplement to address the concerns that were pointed out during the previous round of reviews.

Reviewer #2: I have finished my review of the assigned manuscript. In this study, the authors develop a complex agent-based model to elucidate transmission dynamics of COVID-19. Their model includes unique features and substantial heterogeneity, not found in other compartmental based models.

1) In the agent based model, the location of the agents are given by pixel coordinates. However, it is unclear how many agents can occupy a particular pixel. Is it one agent/pixel? What about the resolution of the epicenters and population densities? Are they at the pixel resolution also?

The geographic system coordinates for epicenters and for those areas of high density are re-coded as Cartesian coordinates that are represented (defined) in pixels. This is done via re-scaling of values and based on the assumption that due to the relatively small modeled geographic region in comparison to the entire planet surface, the discrepancies between the geographic and the Cartesian coordinate systems such as the differences in latitude scales within the region are negligible. The radius R_i (in peters) of each modeled circular epicenter in geographic coordinates is recalculated for the Cartesian coordinate system (in pixels) and is denoted as r_i. After that the location of individuals is generated in the Cartesian coordinate system and represented as pixels on the map. Since the coordinates of each individual are generated randomly via a random radius and via a corresponding angle the coordinates of multiple individuals can indeed match. The process is described in details on pages 4 and 5 of the manuscript supplement. When the individuals are displayed on the map they are represented as small circles that do occupy multiple pixels. When multiple individuals are infected there is an overlap, so the "cloud of points" that represent the individuals are expected to give the boundaries of the infection spread rather than the number of infected individuals. In the web version of the tool there is an option to color-code the "cloud of points" to visualize the density of individuals in each location.

2) The parameters used in the study should be summarized in the table. It is unclear how the natural history of disease progresses for each agent. For example, in the appendix, the authors use a Lognormal distribution to sample the durations of stg {1, 2} in equations 8 and 9, but no reference or justification is given for why these durations were picked. (The authors' reply to another reviewer suggest they indeed included a table, but it should be provided in the main manuscript.)

To address the parameters description we have created and supplied a separate detailed documentation file which have been included together with the tool on the same GitHub shared repository. The repository link is provided in the manuscript (i.e. https://github.com/quantori/COVID19-MA-Transmission). This document contains the requested details description of all parameters and the ways they can be adjusted via GUI. It also provides the information about COVIDSession.txt configuration file the Data folder which is filled by GUI.

The parameters modifications and adjustments by the user are implemented via two mechanisms:

1) For the first mechanism the user can edit those parameters directly within the text file and then open the application. Those manually modified (within the COVIDSession.txt text file) parameter values will be automatically imported into the application on the next open. Then the user has an option to run the program with those values, perform changes within the graphic user interface (GUI) and/or run the optimization procedure.

2) For the second option the parameters within the GUI are saved automatically in COVIDSession.txt file when the user closes the application. In this case COVIDSession.txt contains the last set of parameter values that has been used for the model.

In the beginning we intentionally decided to make those parameter values public and not to put some fixed parameter value into the manuscript/supplement tables. We believe that those parameters can and will be changed/adjusted frequently by the user. This will take place if more inside about their values become available over time or the user wants to investigate multiple values of the same parameter within the model (i.e. sensitivity analysis).

Specifically for equations (8) and (9) the goal was to present a parametrization. During the model calibration we considered multiple possible parametrization and the one presented in details in the Supplement provided the best model fit. There are a lot of different options how to parametrize the process and we presented only one of them. The users are encouraged to make any changes they find necessary provided that they have another preference since the code is publicly available. We presented a method and kept the parametrization question open.

4) Instead of justifying the parameter values used in their study, the authors (correctly) state that they are input values and interested readers have the ability to modify these values in the provided software. There are a few concerns with this:

--- the code is compiled only for Windows, leaving Mac and Linux users unable to run the software (without jumping through hoops such as virtual machines). For these users, the authors should justify their parameters and provide some sensitivity analysis.

In the beginning we intentionally decided to make the parameter values public and not to put them to fixed values. The user can adjust them on the fly. We believe that those parameters can and will be changed/adjusted frequently by the user. This can take place when additional information about the parameter values will become available in the future or the user will decide to investigate multiple values of the same parameter within the model. The parameters are aggregated and summarized in the parameters file COVIDSession.txt within the Data folder.

The parameters modifications and adjustment are implemented via the two options or mechanism. 1) For the first option the user can edit those parameters directly within the text file and then open the application. Those manually modified (within the COVIDSession.txt text file) parameter values will be automatically imported into the application on the next open. Then the user has an option to run the program with those values, perform changes within the graphic user interface (GUI) and/or run the optimization procedure. 2) For the second option the parameters within the GUI and saved automatically in COVIDSession.txt file when the user closes the application after working with it. In this case COVIDSession.txt contains the last set of parameter values that has been used in the model. The described options are available for Windows users as well as for the other platform users (MAC, Linux) via the virtual machines (VM-s). It is important to emphasize that the user does not need to

re-compile the code to easily change the parameters of the model so the realization of the model can be directly used on multiple platforms with the help of VM-s.

--- If the goal is to only provide a framework/software, it should be provided as a web-based tool. Moreover, the narrative of the manuscript needs to be revised to indicate that the work simply provides a tool. As currently written, the narrative is as if they are trying to describe transmission dynamics of COVID-19. For this, the authors definitely need to provide more justification for parameter values.

The goal of our Windows tool development was to present a framework and to illustrate that framework for the real COVID-19 data that were available to the state of Massachusetts. The model was calibrated according to the available Massachusetts data. The code and the model formulations are made freely available such that readers that are interested in the proposed approach have an option to adopt the code to their needs. The provided tool included the windows application and also a limited web interface that is using the tool API in the backend. The source code and the full theory behind the approach is described in full details in the Supplement so the readers can modify the existing code or and adopt the code or write their own adaptation of the model in the language and platform of their choice.

--- The authors should check in a README.md file in their github repository with explicit instructions on compiling and running the software. In addition, all the comments (which describe the flow) are not in English, which makes it difficult to verify/find any possible bugs in the system.

Thank you for your comment. We have created and supplied the detailed readme file in two formats (ReadMe.docx, ReadMe.pdf) which have been included together with the tool on the same GitHub shared repository. The repository link is provided in the manuscript (i.e. https://github.com/quantori/COVID19-MA-Transmission). The readme file contains the requested information and is expected to make the user experience with the tool friendly. It includes the detailed about the tool GUI and about the changes and adjustments of the simulation parameters. It also provides the information about COVIDSession.txt configuration file.

We apologize for the code comments in Russian. We have updated all the source code files comments with English analogues next to each original Russian comment. This will help the readers to follow and to modify the code if needed.

---

6. PLOS authors have the option to publish the peer review history of their article (what does this mean?). If published, this will include your full peer review and any attached files.

If you choose “no”, your identity will remain anonymous but your review may still be made public.

Do you want your identity to be public for this peer review? For information about this choice, including consent withdrawal, please see our Privacy Policy.

Reviewer #1: No

Reviewer #2: No

[NOTE: If reviewer comments were submitted as an attachment file, they will be attached to this email and accessible via the submission site. Please log into your account, locate the manuscript record, and check for the action link "View Attachments". If this link does not appear, there are no attachment files.]

While revising your submission, please upload your figure files to the Preflight Analysis and Conversion Engine (PACE) digital diagnostic tool, https://pacev2.apexcovantage.com/. PACE helps ensure that figures meet PLOS requirements. To use PACE, you must first register as a user. Registration is free. Then, login and navigate to the UPLOAD tab, where you will find detailed instructions on how to use the tool. If you encounter any issues or have any questions when using PACE, please email PLOS at figures@plos.org. Please note that Supporting Information files do not need this step.

In compliance with data protection regulations, you may request that we remove your personal registration details at any time. (Remove my information/details). Please contact the publication office if you have any questions.
