# Supplementary material for: Development of an interactive, agent-based local stochastic model of COVID-19 transmission and evaluation of mitigation strategies illustrated for the state of Massachusetts, USA
Source: PLoS One. 2021 Feb 17;16(2):e0247182. doi: 10.1371/journal.pone.0247182 (PMC7888623; doi:10.1371/journal.pone.0247182)
Supplement: Attachment — Submitted filename: Response_CLEANED.docx [file pone.0247182.s004.docx]

"The authors properly answered my questions in their response letter. My major concern about asymptomatic patients is now mentioned in the main text, with a reference to more details in the supplement, but the supplement was only minimally changed and the word asymptomatic does not even appear in the supplement. Thus some further update on the supplement and maybe on the main text could help to make this complete. The results and the discussion sections are relatively short. It might have been useful to add a section on the limitations of the model as it stands now. The above points are quite properly explained now in the authors' response letter. Maybe some of the comments from here could be moved into the main text or the supplement."

**We have updated both the manuscript and the supplement text with the discussion about the asymptomatic cases.**

**We have also updated the Discussion section of the manuscript where we discussed the model limitations outlined earlier.**

**The changes above are also emphasized in the tracked versions of the manuscript and of the supplement.**

and

"The authors have addressed my concerns, although the flow and narrative of the manuscript can be improved. For example, the authors addressed my comments regarding the lack of a table describing the parameters by including an external link in the manuscript to their code repository. This forces the reader to decipher and navigate the code repository website (github), thus creating an accessibility issue "

**Upon request, we have included an example of the parameters table in the supplement and referred the reader to the online repository for more detailed documentation about the tool.**

**The changes above are also emphasized in the tracked versions of the supplement.**

If you would like to make changes to your financial disclosure, please include your updated statement in your cover letter. Guidelines for resubmitting your figure files are available below the reviewer comments at the end of this letter.

**No changes with the previous submission and re-submission.**

**Comments to the Author**

1. If the authors have adequately addressed your comments raised in a previous round of review and you feel that this manuscript is now acceptable for publication, you may indicate that here to bypass the “Comments to the Author” section, enter your conflict of interest statement in the “Confidential to Editor” section, and submit your "Accept" recommendation.

Reviewer #1: (No Response)

Reviewer #2: All comments have been addressed

2. Is the manuscript technically sound, and do the data support the conclusions?

The manuscript must describe a technically sound piece of scientific research with data that supports the conclusions. Experiments must have been conducted rigorously, with appropriate controls, replication, and sample sizes. The conclusions must be drawn appropriately based on the data presented.

Reviewer #1: Yes

Reviewer #2: Yes

3. Has the statistical analysis been performed appropriately and rigorously?

Reviewer #1: Yes

Reviewer #2: Yes

4. Have the authors made all data underlying the findings in their manuscript fully available?

The [PLOS Data policy](https://nam03.safelinks.protection.outlook.com/?url=http%3A%2F%2Fwww.plosone.org%2Fstatic%2Fpolicies.action%23sharing&data=04%7C01%7Cakirpich%40gsu.edu%7C04838729833145171fc508d8b67485dc%7C515ad73d8d5e4169895c9789dc742a70%7C0%7C0%7C637459958869710002%7CUnknown%7CTWFpbGZsb3d8eyJWIjoiMC4wLjAwMDAiLCJQIjoiV2luMzIiLCJBTiI6Ik1haWwiLCJXVCI6Mn0%3D%7C1000&sdata=1HmtHFGeWhB1SG8sAUs5NFgQ%2F2UG98VuonKTuDICNxQ%3D&reserved=0) requires authors to make all data underlying the findings described in their manuscript fully available without restriction, with rare exception (please refer to the Data Availability Statement in the manuscript PDF file). The data should be provided as part of the manuscript or its supporting information, or deposited to a public repository. For example, in addition to summary statistics, the data points behind means, medians and variance measures should be available. If there are restrictions on publicly sharing data—e.g. participant privacy or use of data from a third party—those must be specified.

Reviewer #1: Yes

Reviewer #2: Yes

5. Is the manuscript presented in an intelligible fashion and written in standard English?

PLOS ONE does not copyedit accepted manuscripts, so the language in submitted articles must be clear, correct, and unambiguous. Any typographical or grammatical errors should be corrected at revision, so please note any specific errors here.

Reviewer #1: Yes

Reviewer #2: Yes

6. Review Comments to the Author

Please use the space provided to explain your answers to the questions above. You may also include additional comments for the author, including concerns about dual publication, research ethics, or publication ethics. (Please upload your review as an attachment if it exceeds 20,000 characters)

Reviewer #1: The authors properly answered my questions in their response letter. My major concern about asymptomatic patients is now mentioned in the main text, with a reference to more details in the supplement, but the supplement was only minimally changed and the word asymptomatic does not even appear in the supplement. Thus some further update on the supplement and maybe on the main text could help to make this complete.
The results and the discussion sections are relatively short. It might have been useful to add a section on the limitations of the model as it stands now.
The above points are quite properly explained now in the authors' response letter. Maybe some of the comments from here could be moved into the main text or the supplement.

**We have updated both the manuscript and the supplement text with the discussion about the asymptomatic cases.**

**We have also updated the Discussion section of the manuscript where we discussed the model limitations outlined earlier.**

**The changes above are also emphasized in the tracked versions of the manuscript and of the supplement.**

Reviewer #2: Dear Authors,

I would like to apologize for the delay in my review. I have found that all my comments have been addressed fully. Thank you.

7. PLOS authors have the option to publish the peer review history of their article ([what does this mean?](https://nam03.safelinks.protection.outlook.com/?url=https%3A%2F%2Fjournals.plos.org%2Fplosone%2Fs%2Feditorial-and-peer-review-process%23loc-peer-review-history&data=04%7C01%7Cakirpich%40gsu.edu%7C04838729833145171fc508d8b67485dc%7C515ad73d8d5e4169895c9789dc742a70%7C0%7C0%7C637459958869710002%7CUnknown%7CTWFpbGZsb3d8eyJWIjoiMC4wLjAwMDAiLCJQIjoiV2luMzIiLCJBTiI6Ik1haWwiLCJXVCI6Mn0%3D%7C1000&sdata=ckshMlM7Oj9YHRS3kk3aCCDl84A2lClk1q1P39xEVFw%3D&reserved=0)). If published, this will include your full peer review and any attached files.


If you choose “no”, your identity will remain anonymous but your review may still be made public.


**Do you want your identity to be public for this peer review?** For information about this choice, including consent withdrawal, please see our [Privacy Policy](https://nam03.safelinks.protection.outlook.com/?url=https%3A%2F%2Fwww.plos.org%2Fprivacy-policy&data=04%7C01%7Cakirpich%40gsu.edu%7C04838729833145171fc508d8b67485dc%7C515ad73d8d5e4169895c9789dc742a70%7C0%7C0%7C637459958869719996%7CUnknown%7CTWFpbGZsb3d8eyJWIjoiMC4wLjAwMDAiLCJQIjoiV2luMzIiLCJBTiI6Ik1haWwiLCJXVCI6Mn0%3D%7C1000&sdata=lMo%2F3bEp0ycNsMEtOKSrKsPNDdRFlWVMgyNcixzcBiU%3D&reserved=0).

Reviewer #1: No

Reviewer #2: No

[NOTE: If reviewer comments were submitted as an attachment file, they will be attached to this email and accessible via the submission site. Please log into your account, locate the manuscript record, and check for the action link "View Attachments". If this link does not appear, there are no attachment files.]

While revising your submission, please upload your figure files to the Preflight Analysis and Conversion Engine (PACE) digital diagnostic tool, [https://pacev2.apexcovantage.com/](https://nam03.safelinks.protection.outlook.com/?url=https%3A%2F%2Fpacev2.apexcovantage.com%2F&data=04%7C01%7Cakirpich%40gsu.edu%7C04838729833145171fc508d8b67485dc%7C515ad73d8d5e4169895c9789dc742a70%7C0%7C0%7C637459958869719996%7CUnknown%7CTWFpbGZsb3d8eyJWIjoiMC4wLjAwMDAiLCJQIjoiV2luMzIiLCJBTiI6Ik1haWwiLCJXVCI6Mn0%3D%7C1000&sdata=NKVT5uzoWeHCOv2Y%2F6XVuBZ0B070K%2BrUd4P1EYljzxQ%3D&reserved=0). PACE helps ensure that figures meet PLOS requirements. To use PACE, you must first register as a user. Registration is free. Then, login and navigate to the UPLOAD tab, where you will find detailed instructions on how to use the tool. If you encounter any issues or have any questions when using PACE, please email PLOS at [figures@plos.org](mailto:figures@plos.org). Please note that Supporting Information files do not need this step.
